# Anti-citrullinated protein antibodies and high levels of rheumatoid factor are associated with systemic bone loss in patients with early untreated rheumatoid arthritis

**DOI:** 10.1186/s13075-016-1116-9

**Published:** 2016-10-06

**Authors:** Serena Bugatti, Laura Bogliolo, Barbara Vitolo, Antonio Manzo, Carlomaurizio Montecucco, Roberto Caporali

**Affiliations:** Rheumatology and Translational Immunology Research Laboratories (LaRIT) and Early Arthritis Clinic, Division of Rheumatology, IRCCS Policlinico San Matteo Foundation/University of Pavia, Piazzale Golgi 19, 27100 Pavia, Italy

**Keywords:** Early rheumatoid arthritis, Anti-citrullinated protein antibodies, Rheumatoid factor, Bone, Osteoporosis

## Abstract

**Background:**

Autoantibodies such as anti-citrullinated protein antibodies (ACPA) are major risk factors for articular bone destruction from the earliest phases of rheumatoid arthritis (RA). The aim of the current study was to determine whether RA-associated autoantibodies also impact on systemic bone loss in patients with early disease.

**Methods:**

Systemic bone mineral density (BMD) was measured in the lumbar spine and the hip in 155 consecutive treatment-naïve patients with early RA (median symptom duration 13 weeks). Demographic and disease-specific parameters, including clinical disease activity, ultrasonographic (US) examination of the hands and wrists, radiographic scoring of joint damage, ACPA and rheumatoid factor (RF) levels were recorded from all patients. Reduced BMD was defined as Z score ≤ -1 SD and analysed in relation to disease-related characteristics and autoantibody subgroups.

**Results:**

Reduced BMD was found in 25.5 % of the patients in the spine and 19.4 % in the hip. Symptom duration, clinical and US disease activity, functional disability and radiographic damage did not significantly impact on spine and hip BMD loss in regression analyses adjusted for possible confounders (age, gender, menopausal status, current smoking, body mass index). In contrast, ACPA positivity (at any level) negatively affected the spine Z-score (adjusted OR (95 % CI) 2.76 (1.19 to 6.42)); the hip Z score was affected by high titres only (adjusted OR (95 % CI) 2.96 (1.15 to 7.66)). The association of ACPA with reduced BMD in the spine was confirmed even at low levels of RF (adjusted OR (95 % CI) 2.65 (1.01 to 7.24)), but was further increased by concomitant high RF (adjusted OR (95 % CI) 3.38 (1.11 to 10.34)). In contrast, Z scores in the hip were significantly reduced only in association with high ACPA and RF (adjusted OR (95 % CI) 4.96 (1.48 to 16.64)).

**Conclusions:**

Systemic BMD in patients with early RA is reduced in relation with ACPA positivity and high RF levels. This finding supports the notion that RA-associated autoimmunity may have a direct causative role in bone remodeling.

## Background

Rheumatoid arthritis (RA) is a chronic immune-inflammatory disease associated with several forms of skeletal remodeling including peri-articular osteopenia, marginal joint erosions and generalised bone loss. Pro-inflammatory cytokines are traditionally regarded as key drivers of articular and extra-articular bone tissue destruction [1–3]. However, recent experimental evidence indicates that RA-associated autoantibodies, in particular anti-citrullinated protein antibodies (ACPA), can independently stimulate bone remodeling by inducing the differentiation of bone-resorbing osteoclasts [4, 5].

Clinically, the association between ACPA and further progression in joint damage has been reported in several independent studies, [6–8]. This association appears at least partially independent of inflammation. Despite having a similar response to steered treatment strategies, ACPA-positive patients with RA indeed have higher rates of joint damage progression over time [9], and serum receptor activator of nuclear factor kappa B ligand (RANKL) is reported to be increased in ACPA-positive patients independent of acute phase reactants and pro-inflammatory cytokines [10]. More intriguingly, elegant imaging studies have recently demonstrated impairment in the bone microstructure in the metacarpophalangeal joints of ACPA-positive healthy individuals despite no signs of arthritis [11].

As ACPA precede the clinical onset of RA by years and are at least initially produced at extra-articular sites [12], it could be expected that ACPA-positive patients with early RA may already show signs of generalised bone loss in addition to destruction in the joints. However, the pathophysiology of secondary osteoporosis in RA is complex and mostly attributed to long-standing, disabling disease [13, 14]. Accordingly, the few studies available in early RA have reported overall bone mineral density (BMD) almost comparable to that of non-RA controls [15–20], and the potential effect of autoantibodies has not been systematically evaluated.

The Pavia Early Arthritis Clinic is a single-centre inception cohort of patients with recent-onset inflammatory arthritis, who are treatment-naïve at inclusion, and who undergo standardised clinical, laboratory and imaging assessments upon referral [21, 22]. Baseline BMD measurement has been systematically introduced since 2012. Here, we took advantage of this cohort to tackle the question of whether RA-associated autoantibodies affect systemic BMD independent of classical demographic and disease-related risk factors in the initial stages of arthritis.

## Methods

### Patients

Between 2012 and 2014, all patients newly referred to the Pavia Early Arthritis Clinic with a diagnosis of RA according to the American College of Rheumatology (ACR)/European League Against Rheumatism (EULAR) 2010 classification criteria [23] were invited to undergo dual-energy x-ray absorptiometry (DXA) at both the hip and the spine. Patients were naïve to glucocorticoids and disease-modifying anti-rheumatic drugs, and had arthritis of short duration (<12 months of symptoms). Patients with definitive diagnoses other than RA, or any suspicion of spondyloarthritis (including personal or familial psoriasis and clinical or imaging evidence enthesitis), were carefully excluded.

Demographic and general characteristics known to affect BMD were obtained by interview and included age, gender, ethnicity, body mass index (BMI), menopausal status, age at menopause, current smoking and alcohol status, risk factors for secondary osteoporosis other than RA, previous clinical fractures, osteoporosis in first-degree relatives, use of calcium and vitamin D supplements, hormone replacement therapy and bisphosphonates (Table 1).

**Table 1:** Demographic and clinical characteristics of the study population

Variable	Value in study participants (*n* = 155)
Demographics	
Age, mean (SD), years	57.8 (13.9)
Female gender, *n* (%)	115 (74.2)
Postmenopausal, *n* (%)	70 (60.9)
Age at menopause, mean (SD), years	49.7 (4.2)
Premature menopause, *n* (%)	3 (4.3)
Caucasian, *n* (%)	148 (95.5)
Body mass index, median (IQR), kg/m^2^	25 (22–28)
Current smoker, *n* (%)	30 (19.4)
Alcohol ≥3 units/day, *n* (%)	3 (1.9)
Comorbidities, mean number (SD)	0.4 (0.6)
Previous fracture, *n* (%)	7 (4.5)
Parent fractured hip, *n* (%)	15 (9.7)
Calcium supplementation, *n* (%)	2 (1.3)
Vitamin D supplementation, *n* (%)	3 (1.9)
Bisphosphonate use, *n* (%)	2 (1.3)
Hormone replacement therapy, *n* (%)	5 (3.2)
Disease characteristics	
Symptom duration, median (IQR), weeks	12.9 (8.6–25.7)
1987 American College of Rheumatology criteria fulfilled, *n* (%)	107 (69)
Disease activity score in 28 joints, mean (SD)	4.42 (1.28)
Swollen joint count in 28 joints, median (IQR)	5 (3–8)
Tender joint count in 28 joints, median (IQR)	6 (3–10)
Ultrasound grayscale score, median (IQR), 0–36 score	5 (2–9)
Ultrasound power Doppler score, median (IQR), 0–36 score	2 (0–7)
Erythrocyte sedimentation rate, median (IQR), mm/1 h	17 (8–33)
C-reactive protein, median (IQR), mg/dlL	0.7 (0.3–1.7)
Rheumatoid factor-positive, *n* (%)	67 (43.2)
Rheumatoid factor titres in positive patients, median (IQR), U/mL	82 (34.8–195.8)
Anti-citrullinated protein antibody-positive, *n* (%)	66 (42.6)
Anti-citrullinated protein antibody titres in positive patients, median (IQR), U/mL	237 (77.5–445.8)
Erosion Sharp–van der Heijde Score ≥1, *n* (%) (*n* = 152)	32 (21.1)
Total Sharp–van der Heijde Score, median (IQR), 0–448 scale (*n* = 152)	3 (1.25–7.5)
Health Assessment Questionnaire, median (IQR), 0–3 scale	1 (0.438–1.438)
DXA	
Spine L1–L4 (*n* = 153)	
Bone mineral density, mean (SD), g/cm^2^	0.936 (0.157)
Z score, mean (SD)	0.013 (1.315)
Z score ≤ -1, *n* (%)	39 (25.5)
- men	13 (33.3)
- premenopausal women	13 (28.9)
- postmenopausal women	13 (18.1)
Total hip (*n* = 155)	
BMD, mean (SD), g/cm^2^	0.728 (0.132)
Z score, mean (SD)	-0.128 (1.027)
Z score ≤ -1, *n* (%)	30 (19.4)
- men	7 (17.5)
- premenopausal women	12 (26.7)
- postmenopausal women	11 (15.7)
Either spine L1–L4 or total hip (*n* = 153)	
Z score ≤1, *n* (%)	54 (35.3)
- men	16 (41)
- premenopausal women	19 (42.2)
- postmenopausal women	19 (27.5)

### Disease variables

Disease duration was defined as the duration of patient self-reported joint symptoms. Disease activity was assessed using the 28-joint disease activity score (DAS28) based on the number of tender and swollen joints, the visual analogue scale for patient’s global assessment (0–100), and the erythrocyte sedimentation rate. C-reactive protein levels were also recorded. IgM rheumatoid factor (RF) and ACPA were determined by immunonephelometry using a Dimension Vista 1500 system (Siemens, Erlangen, Germany) and by a second-generation Phadia ImmunoCAP 250 EliA CCP assay (Phadia, Freiburg, Germany) respectively, according to the manufacturers’ recommendations. A positive result was defined as any value >20 IU/mL for RF and >10 IU/mL for ACPA. A low antibody level was defined as any value greater than the defined upper limit of normal (ULN) and ≤100 IU/ml, and a high antibody level as a value >100 IU/ml [24]. Different thresholds based on the ULN [23] were not tested because of the very low number of patients with ACPA ≤3 ULN (≤30 IU/mL according to our assay).

Functional ability was measured by the Health Assessment Questionnaire Disability Index. Ultrasound (US) examination was performed by a single experienced operator using a Logiq 9 scanner (General Electrics Medical Systems) with a multifrequency linear array transducer (8–15 MHz), according to EULAR guidelines [25]. The US assessment included transverse and longitudinal scanning of the medial and lateral dorsal aspects of both wrists and the first to fifth metacarpophalangeal joints, as previously described [21, 22]. Grayscale (GS) and power Doppler (PD) signals were assigned to each joint in accordance with semi-quantitative 0–3 scales, and an overall US score for GS and PD was calculated as the sum of either GS or PD signal scores obtained from each joint (range 0–36). Radiographic joint damage according to the Sharp–van der Heijde score (SHS) [26] was rated independently by two experienced physicians, and the mean of the scores of the two assessors was used for analysis.

### BMD measurements

Out of 167 eligible patients, 155 agreed to undergo BMD measurement, with no significant differences between those who did or did not consent (not shown). BMD measurements in the left hip (femoral neck and total hip) and lumbar spine, vertebrae L1–4 were performed using the same DXA equipment (Hologic, Vilvoorde, Belgium). All procedures were performed by two trained technicians in accordance with the manufacturer’s standardised procedures. Due to logistic reasons, spine measurement was not performed in two patients. Z score estimations were computed through age-adjusted and gender-adjusted BMD according to locally used reference populations provided by the manufacturer. The Z score was used in order to correct for the heterogeneous age and gender of our cohort and to directly estimate the contribution of RA-related factors beyond classical determinants of primary osteoporosis [27, 28]. Reduced BMD was defined as a Z score ≤ -1 SD [13, 14, 20].

### Statistical analyses

Data were described as mean and standard deviation (SD) or median and interquartile range (IQR) if continuous and as counts and percent if categorical. Disease-related variables in relation to the occurrence of reduced BMD (Z score ≤ -1 SD) were analysed by regression analyses adjusted for possible confounders (age, gender, menopausal status, current smoking and BMI). Comparisons between autoantibody subgroups were evaluated using the independent samples *t* test or analysis of variance (ANOVA) with Bonferroni post hoc testing. Statistical analyses were performed using MedCalc® Version 12.7.0.0, and the level of significance was set at 0.05.

## Results

### Characteristics of the study population

Table 1 shows the characteristics of the 155 patients with early RA for whom baseline BMD was available. The study population mainly consisted of women (74.2 %), of whom 60.9 % were postmenopausal. Mean (SD) age was 58 (14) years, and 76.1 % of the subjects were <70 years of age.

Patients were treatment-naïve with a short history of RA (median (IQR) 12.9 (8.6–25.7) weeks). Sixty-nine percent also fulfilled the 1987 ACR classification criteria for RA [29]. All had active disease (mean (SD) DAS28 4.42 (1.28)), 64.8 % had PD-positive synovitis (PD score >0), and 21.1 % had evidence of radiographic erosions (erosion SHS >0). Sixty-seven patients (43.2 %) were RF-positive, 66 (42.6 %) were ACPA-positive, and 58 (37.4 %) were double-positive.

### Prevalence and distribution of reduced BMD

Mean (SD) Z scores were 0.01 ± 1.31 in the spine and -0.13 ± 1.03 in the hip. BMD was below the expected range for gender and age (Z score ≤ -1) in the spine in 25.5 % of the patients, in the hip in 19.4 % of patients, and in either site in 35.3 % of patients. Reduced BMD tended to be more common in men and premenopausal women in the spine, and in premenopausal women in the hip (Table 1).

### BMD in relation to disease duration and activity

**Fig. 1:**
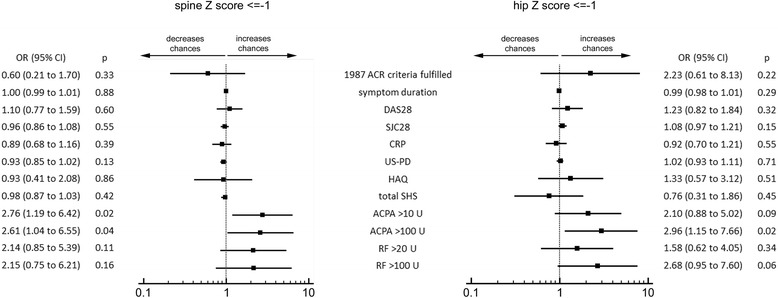
Systemic bone mineral density in relation to disease-related variables. Regression analysis of reduced bone mineral density (Z score ≤ -1) in the spine and the hip. Odds ratios (*OR*) are presented with corresponding 95 % CI levels. All variables were adjusted for age, gender, menopausal status, body mass index and current smoking. *ACR* American College of Rheumatology, *DAS28* 28-joint disease activity score, *SJC28* swollen joint count in 28 joints, *CRP* C-reactive protein, *US-PD* ultrasonographic power Doppler score, *HAQ* Health Assessment Questionnaire, *SHS* Sharp–van der Heijde score, *ACPA* anti-citrullinated protein antibodies, *RF* rheumatoid factor

The relationship between reduced BMD and disease variables is shown in Fig. 1. Fulfillment of the 1987 ACR criteria, symptom duration and clinical, laboratory or imaging parameters of inflammation were not significantly associated with Z score ≤ -1 in the measured sites. Similarly, the very low levels of functional disability and radiographic damage characterising our cohort did not appear to impact on systemic BMD.

### BMD in relation to autoantibody positivity and levels

ACPA-positive patients had significantly lower Z scores in the spine compared to ACPA-negative patients (mean -0.35 ± 1.19 vs 0.30 ± 1.34, *p* = 0.003), and ACPA emerged as independent predictors of reduced BMD with an adjusted OR (95 % CI) of 2.76 (1.19–6.42) (Fig. 1). The difference in hip Z scores was borderline significant (mean -0.28 ± 1.03 vs 0.00 ± 1.00, *p* = 0.09), whilst RF per se did not impact on either spine or hip BMD loss (adjusted ORs (95 % CI) 2.14 (0.85 to 5.39) and 1.58 (0.62 to 4.05) respectively) (Fig. 1).

**Fig. 2:**
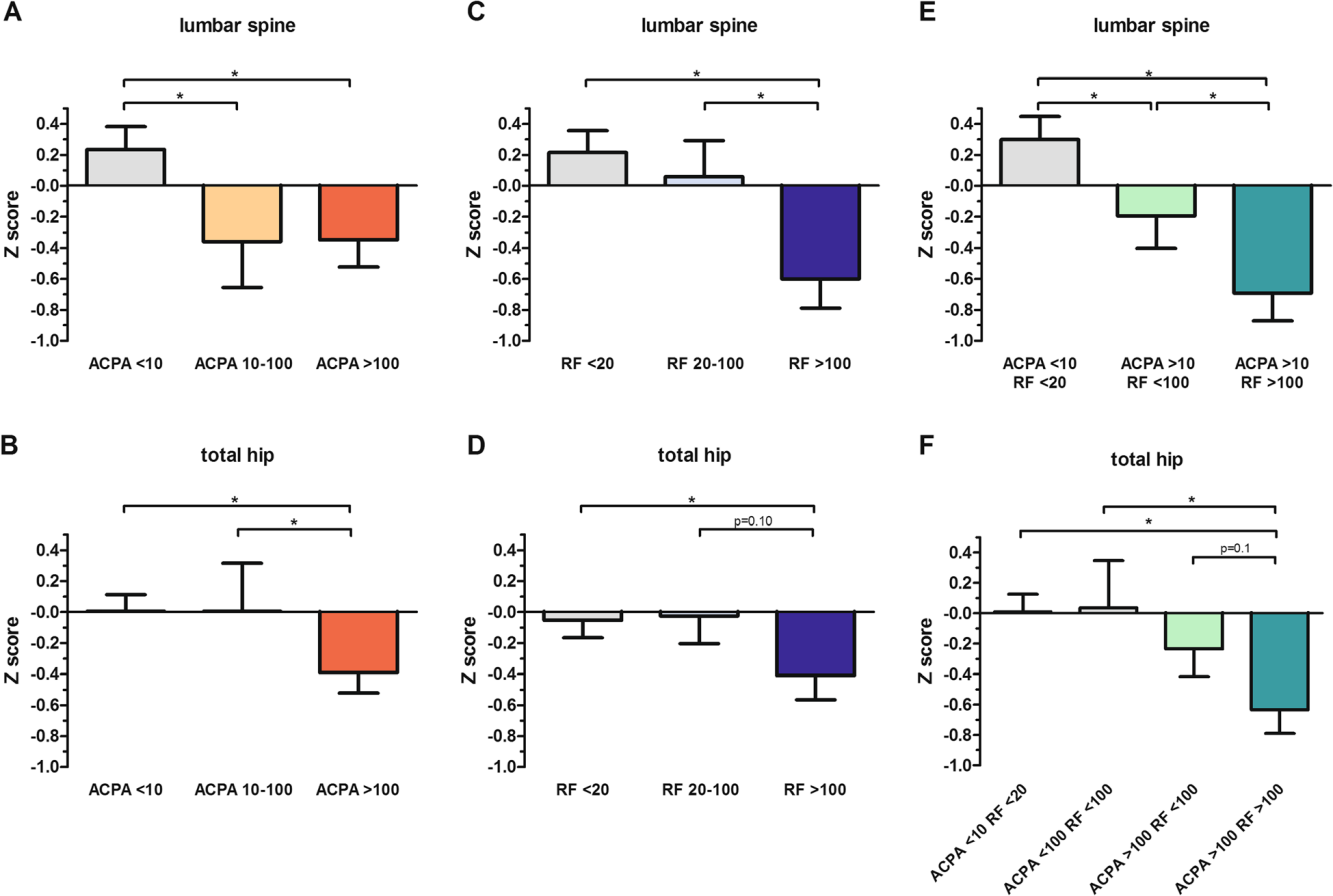
Association between systemic bone mineral density and anti-citrullinated protein antibodies and/or rheumatoid factor. Mean Z scores in the lumbar spine, vertebrae L1-L4 (**a**, **c**, **e**) and total hip (**b**, **d**, **f**) in subgroups of patients stratified according to anti-citrullinated protein antibody (*ACPA*) titres (**a**, **b**), rheumatoid factor (*RF*) titres (**c**, **d**) and the combination of ACPA and RF (**e**, **f**). *Significant differences (*p* <0.05)

We further questioned whether ACPA and RF levels (rather than dichotomous positivity) had different effects on BMD, and whether there is any interaction between the two autoantibody systems. We thus compared Z scores in patients with negative ACPA (<10 U), and in those with low (10–100 U) and high (>100 U) values [24]. On ANOVA, these subgroups differed significantly in both the spine and the hip (Fig. 2a, b). For spine Z scores, differences between ACPA-positive patients with low and high values were not significant, and both subgroups had similarly reduced BMD compared with ACPA-negative patients (Fig. 2a). In contrast, reductions in Z scores in the hip were restricted to patients with high ACPA values (Fig. 2b), and ACPA >100 U predicted hip BMD loss with an adjusted OR (95 % CI) of 2.96 (1.15–7.66) (Fig. 1). The relationship between RF and BMD loss was instead clearly dose-dependent in both the spine and the hip, with significant differences being observed for patients with high values only (Fig. 2c, d).

When combining ACPA with RF titres, it was evident that the presence of ACPA negatively affected the Z score in the spine even in the presence of low levels of RF (difference in means (Δmeans) 0.50, SE 0.26 compared to seronegative patients), but RF >100 U conferred additional risk (Δmeans 0.50, SE 0.30 compared to patients who were ACPA-positive RF-low) (Fig. 2e). Accordingly, the adjusted OR (95 % CI) of ACPA in predicting spine Z score ≤ -1 increased from 2.65 (1.01–7.24) in the presence of concomitant RF ≤100 to 3.38 (1.11–10.34) for RF >100. In contrast, Z scores in the hip were significantly reduced only in association with high ACPA and RF. (Fig. 2f). ACPA >100 and RF >100 predicted reduced BMD in the hip with an adjusted OR (95 %) of 4.96 (1.48–16.64).

## Discussion

We show here that, overall, systemic BMD is preserved in patients with early RA, and classic disease-related risk factors, including disease duration, inflammatory activity and functional ability, have negligible impact on measurable bone remodeling at this stage of the disease. However, ACPA appear associated with significantly reduced BMD, and the concomitant presence of high levels of RF further enhances the risk of bone loss. Whilst the effect of ACPA on the trabecular bone of the spine is readily appreciable, changes in the cortical bone of the hip require higher levels of autoantibodies.

The small proportion of patients with reduced BMD in our study is consistent with earlier reports [15–20] and confirms that overt secondary osteoporosis in RA mostly develops in association with chronic, disabling disease. The short symptom duration and the low levels of functional impairment and radiographic damage in our contemporary cohort diagnosed according to the 2010 criteria may explain the lack of association between disease-related factors and BMD, as compared with historical groups of patients with early RA with longer disease duration and variable treatment [15, 19, 20].

Whilst synovial inflammation may thus take longer to produce systemic effects at joint-remote sites [30], we show here that RA-associated autoimmunity appears coupled with reduced BMD from the early stages of clinical disease. Although functional verification of the pathogenic effects of autoantibodies on extra-articular bone in our study is lacking, our data fit with recent evidence linking ACPA to bone changes in vitro and in vivo [4–6]. Relevantly, the association between ACPA and BMD loss was enhanced by high levels of RF. Sensitive imaging techniques have shown that RF dose-dependently influences the size of bone erosions on an ACPA background [24], and the combined presence of ACPA and RF mediates increased pro-inflammatory cytokine production in vitro [31, 32]. The immune-complex activity of RF may have thus elicited a subclinical inflammatory milieu able to enhance ACPA-mediated osteoclast activation also at joint-remote sites. The different associations between autoantibody levels and spine and hip BMD loss might be explained by the higher rate of turnover in trabecular compared to cortical bone [33].

Our study has some limitations. Although we cannot exclude RA over-diagnosis according to the 2010 criteria [34], differential diagnoses were carefully evaluated and applying the 1987 criteria did not affect the results. The frequency of autoantibodies was lower than expected [35–37], but still comparable with other early arthritis cohorts from similar geographical areas [38], including the *Etude et Suivi des POlyarthrites Indifférenciées Récentes* (ESPOIR) cohort, in which 46–49 % of the patients fulfilling the RA classification criteria at inclusion are reported as ACPA-positive [39–41]. Irrespective of the prevalence of autoantibodies, however, we believe that the observed differences in BMD according to autoantibody levels clearly confirm the specific association between RA autoimmunity and systemic bone loss. Although the small number of single-positive patients hampers definitive conclusions on the independent associations with ACPA and RF, spine BMD was significantly reduced in ACPA-positive patients with low RF (Fig. 1e) in the absence of the definitive effects of low RF (Fig. 1c). This finding indirectly supports the notion that ACPA are key drivers of bone damage, and RF becomes important when ACPA are also present [24]. Also, the cross-sectional character of this study hampers definition of the potential effect of autoantibodies on the progression of BMD loss. DXA follow up is ongoing to define whether treatment can halt autoantibody-dependent systemic bone remodeling. It is also equally important to emphasise that additional measures of bone quality, including micro-architecture, mineralisation and turnover [42–44], might help better define the net impact of autoantibodies and autoantibody levels on bone. Finally, it is important to recall that vitamin-D is a key determinant of bone health [45], and patients with RA have significantly lower vitamin D serum levels compared to healthy controls [46]. As vitamin D deficiency might be more prevalent in ACPA-positive patients [47], we cannot exclude that possibility that the observed association between autoantibodies and reduced BMD might be at least partly mediated by lower vitamin D levels.

## Conclusions

In summary, our data suggest that ACPA are associated with systemic bone loss from the earliest stages of RA, and high levels of RF further increase the risk. ACPA-RF-positive patients with early RA should thus be carefully monitored for the development of generalised osteoporosis beyond the assessment of traditional risk factors.

## Abbreviations

ACPA: Anti-citrullinated protein antibodies
ACR: American College of Rheumatology
ANOVA: Analysis of variance
BMD: Bone mineral density
BMI: Body mass index
CI: Confidence interval
DAS28: 28-joint disease activity score
DXA: Dual-energy x-ray absorptiometry
EULAR: European League Against Rheumatism
GS: Grayscale
IQR: Interquartile range
OR: Odds ratio
PD: Power Doppler
RA: Rheumatoid arthritis
RF: Rheumatoid factor
SD: Standard deviation
SHS: Sharp–van der Heijde score
ULN: Upper limit of normal
US: Ultrasonography
